# Impact of Sacubitril/ Valsartan on quality of life and ejection fraction of heart failure patients with and without chronic kidney disease

**DOI:** 10.12669/pjms.40.6.7892

**Published:** 2024-07

**Authors:** Syeda Huma Zartash, Sidra Saleem, Abeera Mansur, Zain Rasool, Shahryar Ahmad Sheikh

**Affiliations:** 1 Syeda Huma Zartash, MBBS, MRCP. Doctors Hospital & Medical Center, Lahore, Pakistan; 2 Sidra Saleem, MBBS, FCPS, MRCP. Doctors Hospital & Medical Center, Lahore, Pakistan; 3 Abeera Mansur, MBBS, MD, FACP, FASN. Doctors Hospital & Medical Center, Lahore, Pakistan; 4 Zain Rasool, MBBS. Doctors Hospital & Medical Center, Lahore, Pakistan; 5 Shahryar Ahmad Sheikh, MBBS, M.D, FACC. Doctors Hospital & Medical Center, Lahore, Pakistan

**Keywords:** Sacubitril, Valsartan, Heart failure, Chronic Kidney disease, Quality of Life

## Abstract

**Objective:**

Chronic kidney disease (CKD) patients are at high risk of heart failure (HF) and both share similar risk factors, including diabetes and elevated blood Pressure (B.P). Aim of this study was to determine the impact of sacubitril/valsartan on the quality of life (QOL) and ejection fraction (EF) of patients with HF with and without CKD.

**Methods:**

Single center (Doctors Hospital Lahore), observational study with longitudinal follow up, on 104 HF patients from July 2019 to July 2020. HF was diagnosed on both clinical and echo parameters. New York Heart Association Class II-IV, EF less than or equal to 40% HF with reduced EF and stage three CKD patients were included. Sacubitril/Valsartan was prescribed at a starting daily dose of 50mg and then up titrated to 400mg. Patients were followed up with clinical evaluation, QOL assessment, echocardiography and biochemical profile at one, four, eight and 12 months.

**Results:**

Gender, age, and diabetes mellitus between CKD and non-CKD patients were noted to be statistically different, defined as p<0.05. CKD patients’ QOL increased from 45.15 to 57.57 from baseline to 12 months (p-value<0.01). Non-CKD patients’ QOL increased from 48.07 to 56.25. In CKD patients, EF increased from 27.87% to 29.29% from baseline to 12 months (p-value 0.03) whereas in non-CKD patients EF improved from 29.42% to 31.43%.

**Conclusion:**

Sacubitril/ valsartan improves QOL in patients of HF with reduced EF both with and without CKD. Clinical improvement was independent of Left Ventricular EF as measured by QOL. Thus, QOL is a useful tool to assess the drug’s beneficial effect.

## INTRODUCTION

Heart failure (HF) is a considerable global public health problem emerging as an epidemic in developing countries.^1^-^3^ Patients with chronic heart failure continue to be at high risk for HF progression, decreased quality of life, and increased mortality despite considerable therapeutic advancements. In these patients, neurohormonal activation is playing an important role in the development and advancement of the disease.^4^-^6^

A first-in-class drug called sacubitril/valsartan comprises the neprilysin (NEP) inhibitor (sacubitril) and the angiotensin-II (Ang-II) receptor blocker (valsartan). Natriuretic peptides, bradykinin, and Ang-II are among the vasoactive peptides that are metabolized by the endopeptidase.^7^ As a result, its blockage raises levels of Ang-II, whose effects are countered by the angiotensin receptor blocker valsartan, as well as natriuretic peptides, which promote diuresis, natriuresis, and vasodilation.^8^-^10^

Patients with Chronic Kidney disease (CKD) is at high risk of heart failure and both these conditions share similar risk factors, including diabetes and elevated blood pressure. The pathophysiology between the heart and the kidneys is intricate and reciprocal. Traditional HF risk factors and kidney-specific risk factors, such as malnutrition, acid-base disturbances, uremic toxins, changes in bone mineral composition, anemia, and myocardial shock, are more common in patients with CKD.^11^ Preventing disease progression (mortality, hospitalizations, and degradation of left ventricular function) and relieving patients’ suffering are the two main objectives of heart failure care.

## METHODS

This is a single center, observational study with longitudinal follow up, on 104 consecutive heart failure patients (at Doctor’s Hospital and Medical center, Lahore), from July 2019 to July 2020. Written informed consent was obtained from all patients.

### Ethical Approval

Ethical and institutional review board approval was obtained prior to enrolment of patients with IRB approval number: IRB/06/2018/01. Dated: 29^th^ June,2019.

### Inclusion Criteria:

- Patients with heart failure (NYHA class II-IV).
- LV EF(less than or equal to 40%).
- eGFR (more than 30 ml/min /1.73m2).

### Exclusion criteria:

- Symptomatic hypotension.
- eGFR <30 ml/min/1.73m2.
- Serum potassium >5.2mmol/L.
- Angioedema.

Heart failure was diagnosed on both clinical and echocardiographic parameters. NYHA (II-IV) patients with 104 HF presenting to OPD’s and CCU of Doctor’s Hospital and Medical Centre, who fulfilled the inclusion criteria and started on sacubitril /valsartan, were enrolled in study after an informed consent. All the information was entered on proformas. All patients were prescribed Sacubitril/Valsartan at a total daily dose of 50mg and then up titrated to goal dose of 400 mg according to blood pressure tolerance. Both naive patients and those previously on ACE-I and ARB were included after a window period of 48 hours. Quality of life assessment was done according to Kansas City Cardiomyopathy Questionnaire 12, at the start and at intervals of 1st month, 4th month, 8th month and 12th month.^12^

All patients were followed up with serum creatinine, eGFR, serum potassium and blood pressure monitoring at 2/4 weeks, 4^th^ months, 8^th^ months and 12^th^ month. Echocardiography for LV EF was done at start,1st month, 4th month, 8th month and 12th month. CKD was labelled according to KDIGO guidelines.^13^ Data was stored and analyzed using IBM-SPSS version 25. Counts with percentages were reported for qualitative sets of variables and means with standard deviation were given for quantitative data sets. Comparison of baseline characteristics of samples between CKD and Non-CKD samples was done using Pearson Chi Square test. Effect of medication on blood pressure, ejection fraction, potassium, creatinine, eGFR and quality of life from baseline to last 12th month of study was analyzed using Paired sample t-test among CKD and non-CKD patients separately. P-values less than 0.05 were considered statistically significant.

## RESULTS

Among CKD patients (n=46), 58.7% were males, mean age was 62.8 (SD=±9.9) years. 30(68.2%) were diabetic, 26(59.1%) were hypertensive and ischemic heart disease was noted in 43(95.6%) (Coronary artery bypass grafting done in 28.9% and percutaneous coronary intervention (PCI) performed in 28.9%).

Among non-CKD patients (n=57), 82.5% were males with a mean age of 55.4 (SD=±11) years. 27(47.4%) were diabetic, 29 (50.9%) were hypertensive, and 49(86%) had ischemic heart disease (17.5% had undergone coronary artery bypass grafting, and 21.1% had PCI). Gender, age, and diabetes mellitus between CKD and non CKD patients were noted to be statistically different defined as p<0.05 as shown in Table-I.

**Table-I:** Baseline Characteristics of Studied Samples.

Characteristics		Total		Non CKD (n=57)		CKD Stage 3 (n=46)		p-value

		n	%	n	%	n	%	
Gender	Female	29	27.9	10	17.5	19	41.3	<0.01*
	Male	75	72.1	47	82.5	27	58.7	
Mode of admission	OPD	80	76.9	46	80.7	33	71.7	0.28
	Inpatient	24	23.1	11	19.3	13	28.3	
Age (years)	Mean ±SD	58.7	±11.1	55.4	±11.0	62.8	±9.9	<0.01*
Diabetes mellitus	Yes	58	56.9	27	47.4	30	68.2	0.03*
Hypertension	Yes	56	54.9	29	50.9	26	59.1	0.41
Ischemic heart disease	Yes	93	90.3	49	86.0	43	95.6	0.10
Coronary artery bypass Graft	Yes	24	23.3	10	17.5	13	28.9	0.17
Percutaneous coronary intervention	Yes	25	24.3	12	21.1	13	28.9	0.36
eGFR(ml/min)	Mean ±SD	67.24	±25.69	83.5	±22.8	47.0	±9.3	<0.01*

* p<0.05 was considered significant.

**Fig.1:**
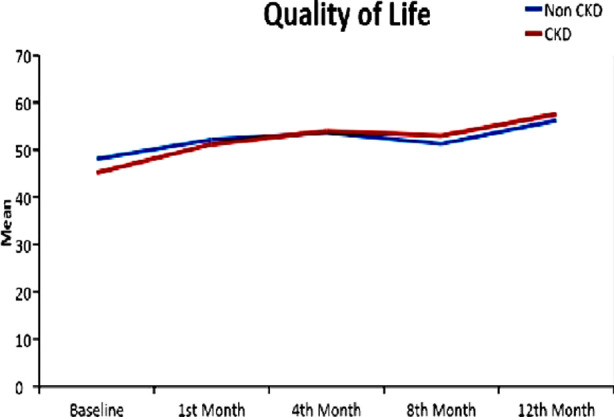
Mean comparison of QOL between CKD and non CKD patients.

**Fig.2:**
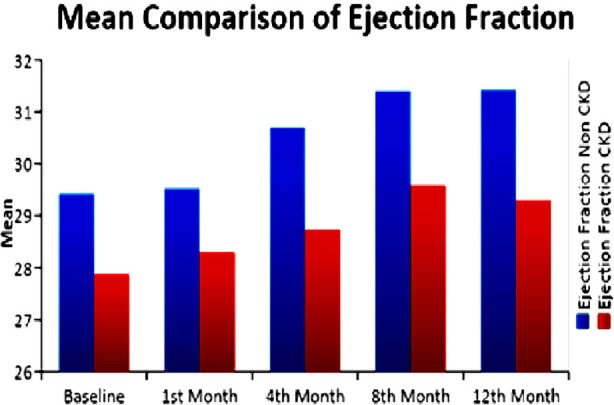
Mean comparison of Ejection Fraction.

In all patients, QOL increased from an average score of 46.91 at baseline to 56.86 at 12 months with p-value <0.01. In the CKD patients, QOL increased from 45.15 to 57.57 from baseline to 12 months (p-value<0.01) with an average increase of 12.42 units. In the non-CKD patients, QOL increased from 48.07 to 56.25 from baseline to 12 months (p-value<0.01) with an average increase of 8.18 units. There was no significant difference in improvement of mean QOL between CKD and non CKD patients as shown in Fig.1. Maximum improvement in QOL was seen at six to eight months after starting the drug in all patients, after which it plateaus. The mean and standard deviation of studied variables in CKD and non CKD patients from baseline to 12^th^ month of study is shown in Table-II. It was observed that ejection fraction increased on average 2.01 units in non CKD group with p-value of 0.01. Among CKD samples, however ejection fraction increased on average 1.42 units, with p-value of 0.03, as shown in Fig.2. eGFR showed an improvement of 5.84ml/min in the CKD group with a p-value of < 0.01.

**Table-II:** Comparison of Studied Parameters in Non-CKD and CKD Patients.

All patients	Baseline	1^st^ Month	4^th^ Month	8^th^ Month	12^th^ Month	Mean difference	p-value
Quality of life	46.91±12.3	51.84±9.99	53.86±9.79	52.05±7.51	56.86±7.85	9.44	<0.01*

*Non CKD Patients*	*Baseline*	*1^st^ Month*	*4^th^ Month*	*8^th^ Month*	*12^th^ Month*	*Mean Difference*	*p-value*

	*Mean±SD*	*Mean±SD*	*Mean±SD*	*Mean±SD*	*Mean±SD*		

Systolic BP(mmHg)	118±15	118±14	119±14	119±14	120±14	1.05	0.057
Diastolic BP(mmHg)	75±9	75±8	76±8	76±8	76±8	0.83	0.10
Ejection Fraction (%)	29.42±7.01	29.54±7.03	30.7±8.63	31.4±9.28	31.43±9.72	2.01	<0.01*
Creatinine(mg/dl)	0.98±-0.26	1.02±0.22	1.01±0.22	1.01±0.21	1.04±0.29	0.06	0.09
eGFR(ml/min)	83.5±22.8	81.2±22.0	81.8±22.4	81.2±22.4	81.0±24.1	-3.28	0.30
Quality of Life	48.07±11.31	52.19±9.52	53.63±9.59	51.31±8.4	56.25±8.86	8.18	<0.01*

*CKD Patients*	*Baseline*	*1^st^ Month*	*4^th^ Month*	*8^th^ Month*	*12^th^ onth*	*Mean Difference*	*p-value*

	*Mean±SD*	*Mean±SD*	*Mean±SD*	*Mean±SD*	*Mean±SD*		

Systolic BP(mm Hg)	122±16	120±16	120±14	120±14	121±14	-1.14	0.50
Diastolic BP(mm Hg)	75±8	74±8	75±8	75±8	75±8	0.14	0.84
Ejection Fraction (%)	27.87±6.23	28.3±6.39	28.72±6.48	29.59±6.13	29.29±6.04	1.42	0.03*
Creatinine(mg/dl)	1.48±0.36	1.44±0.41	1.40±0.37	1.39±0.40	1.39±0.42	-0.10	<0.01*
eGFR(ml/min)	46.0±9.4	52.9±16.3	51.0±12.3	52.7±14.8	53.4±16.2	5.84	<0.01*
Quality of Life	45.15±13.32	51.18±10.64	53.98±10.19	53±5.97	57.57±6.34	12.42	<0.01*

* p<0.05 was considered statistically significant using Paired sample t-test.

## DISCUSSION

Several studies have been performed to assess impact of sacubitril valsartan on quality of life of heart failure patients. However, assessment of quality of life in heart failure patients with CKD is not well studied especially in Southeast Asia. Our study signifies the importance of quality of life as a cost-effective tool to assess drug’s beneficial effect since cost is a barrier in doing various investigations in third world countries.

CKD patients had an increased mean age and increased prevalence of diabetes, hypertension and IHD as compared to non CKD group. In line with our study, many studies show a year.^14^ Heart failure badly effects quality of life of patients. A locally conducted study on 95 heart failure patients showed poor sleep quality and depression in all patients.^15^

In our study quality of life improved by 12.42 units in CKD patients and 8.18 units in non CKD patients at 12 months. Likewise, a study done on 678 HFrEF patients showed that following sacubitril/valsartan initiation, 60.8% of participants experienced a rise in KCCQ-23 by10 points and 26.0% by 20 points.^16^ Another local study done on 80 patients showed significant improvement in functional class after 12 weeks of initiation of sacubitril valsartan.^17^

Similarly, PARADIGM-HF trial, noted improvements in both KCCQ clinical summary score (+0.64 versus -0.29; P=0.008) and KCCQ overall summary score (+1.13 versus -0.14; P<0.001) in sacubitril valsartan group as compared to enalapril group at 8 months.^18^ A secondary analysis of Paradigm HF trial^19^ reported the largest improvement noted in house hold chores and sexual relationships at 36 months.

Another interesting study included 35 non-responders to cardiac resynchronization therapy (75 ± 7 years, 28% females, mean left ventricular ejection fraction 28 ± 8%, 54% non-ischemic cardiomyopathy) with maximally optimized drug therapy (ACE/ARB) and New York Heart Association Class II-III. Patients were then prescribed sacubitril/valsartan and it drastically increased QOL, decreased physical limitations and the number of hospitalizations.^20^

Our study shows an improved ejection fraction over a span of a year. The percentage improvement though, not very significant, yet it had a significant p-value of less than 0.05 in both study cohorts. Many studies support reverse remodeling of LV and improvement of LV volumes, mass and systo-diastolic function.^21^,^22^

Since the publication of PARADIGM, other studies have appeared that also corroborate a lower reduction in the GFR with this drug.^23^-^25^ However, clinical evidence was still lacking for patients with advanced CKD (stage 3b-4), who are commonly excluded from most clinical trials. We found an improvement in renal function in our CKD cohort after initiating the treatment.

### Limitations

It includes small sample size. Enrolments were prematurely curtailed due to Covid-19 related pandemic and logistical limitations in patient follow ups. Other limitations are lack of a control group and inclusion of CKD three patients. We need more studies with eGFR less than 30 ml/minutes..

## CONCLUSION

Sacubitril/ valsartan improves QOL in patients of HFrEF both with and without CKD. Clinical improvement was independent of LVEF as measured by QOL. Thus, QOL is a useful tool to assess the drug’s beneficial effect.

### Author’s contributions:

**SHZ**, **SS**, **AM**, **ZR** and **SAS:** Has made substantial contribution in conception and design of study, drafting of manuscript, revising and editing the manuscript critically for intellectual content. All has approved the submitted version.

**SHZ:** Data preparation and presentation, research coordination and management and is responsible and accountable for the accuracy and integrity of the work.
